# KMT2D regulates specific programs in heart development via histone H3 lysine 4 di-methylation

**DOI:** 10.1242/dev.132688

**Published:** 2016-03-01

**Authors:** Siang-Yun Ang, Alec Uebersohn, C. Ian Spencer, Yu Huang, Ji-Eun Lee, Kai Ge, Benoit G. Bruneau

**Affiliations:** 1 Gladstone Institute of Cardiovascular Disease, San Francisco, CA 94158, USA; 2 Roddenberry Center for Stem Cell Biology and Medicine at Gladstone, San Francisco, CA 94158, USA; 3 Biomedical Sciences Graduate Program, University of California, San Francisco, San Francisco, CA 94158, USA; 4 National Institute of Diabetes and Digestive and Kidney Diseases, National Institutes of Health, Bethesda, MD 20892, USA; 5 Cardiovascular Research Institute, University of California, San Francisco, San Francisco, CA 94143, USA; 6 Department of Pediatrics, University of California, San Francisco, San Francisco, CA 94143, USA

**Keywords:** KMT2D, MLL2, MLL4, ALR, Kabuki syndrome, H3K4 methylation, Heart development, Mouse

## Abstract

*KMT2D*, which encodes a histone H3K4 methyltransferase, has been implicated in human congenital heart disease in the context of Kabuki syndrome. However, its role in heart development is not understood. Here, we demonstrate a requirement for KMT2D in cardiac precursors and cardiomyocytes during cardiogenesis in mice. Gene expression analysis revealed downregulation of ion transport and cell cycle genes, leading to altered calcium handling and cell cycle defects. We further determined that myocardial *Kmt2d* deletion led to decreased H3K4me1 and H3K4me2 at enhancers and promoters. Finally, we identified KMT2D-bound regions in cardiomyocytes, of which a subset was associated with decreased gene expression and decreased H3K4me2 in mutant hearts. This subset included genes related to ion transport, hypoxia-reoxygenation and cell cycle regulation, suggesting that KMT2D is important for these processes. Our findings indicate that KMT2D is essential for regulating cardiac gene expression during heart development primarily via H3K4 di-methylation.

## Abstract (precis)

**Highlighted article:** Cardiac-specific depletion of the H3K4 methyltransferase KMT2D causes dysregulation of genes associated with cell cycle regulation, ion homeostasis and hypoxia signaling.

## INTRODUCTION

During heart development genes are tightly regulated to ensure expression in specific cardiac tissues at the appropriate time. It has emerged that dynamic changes in chromatin structure are crucial in controlling cardiac gene expression, implicating several chromatin remodelers and histone-modifying enzymes in the regulation of heart development, although the precise role of many chromatin modifiers remains unknown (Chang and Bruneau, 2012).

H3K4 methylation is a histone modification linked to transcriptional activation. H3K4me1, along with H3K27Ac enrichment, is associated with active enhancers (Creyghton et al., 2010), and H3K4me3 is highly enriched at promoters (Lauberth et al., 2013), whereas H3K4me2 is enriched at enhancers, promoters and within the gene body (Bernstein et al., 2005; Pekowska et al., 2010). Each histone mark is associated with specific regulatory elements and functions, indicating a complex control of active gene transcription.

A study of *de novo* mutations in severe congenital heart defect (CHD) cases showed a significant over-representation of genes related to H3K4 methylation (Zaidi et al., 2013), highlighting the importance of this histone modification in heart development. In particular, mutations in the H3K4 methyltransferase *KMT2D* (also known as *MLL2*, *MLL4* and *ALR*) have been identified as a major cause of Kabuki syndrome, with ∼60% of patients diagnosed with CHDs, most frequently aortic coarctation, atrial and ventricular septal defects. Most *KMT2D* mutations are predicted to result in haploinsufficiency (Ng et al., 2010; Matsumoto and Niikawa, 2003), suggesting an important role for *KMT2D* in heart development (Digilio et al., 2001; Yuan, 2013; Ng et al., 2010).

KMT2D is a key regulator of gene expression in the context of cellular differentiation in diverse tissues. Hu et al. (2013), Guo et al. (2013) and Lee et al. (2013) showed reduced global H3K4me1 levels in a *KMT2C/D* (*MLL3/4*) double-knockout colon cancer cell line, and identified a majority of KMT2D binding sites located in putative enhancer elements. *Kmt2d* is essential for mouse adipogenesis, myogenesis, macrophage activation and lymphomagenesis, demonstrating additional roles for KMT2D as a mono- and di-methyltransferase at enhancers (Lee et al., 2013; Kaikkonen et al., 2013; Ortega-Molina et al., 2015). Collectively, these findings indicate that KMT2D regulates key gene expression programs via H3K4 mono- and di-methylation, and its role depends on cellular and temporal contexts.

In the present study, we identify KMT2D as an essential regulator of heart development. A single copy of *Kmt2d* is sufficient for normal heart development and leads to mild alterations in heart function. *Kmt2d* deletion in cardiac precursors and cardiomyocytes disrupts cardiogenesis. We show that *Kmt2d* deletion in these cardiac populations results in downregulation of ion transport and cell cycle genes, leading to altered calcium handling and cell cycle defects in cardiomyocytes. Myocardial deletion of *Kmt2d* leads to decreased H3K4me1 at H3K27Ac-enriched enhancers and decreased H3K4me2 at promoters and enhancers. Finally, we identify KMT2D binding regions in cardiomyocytes, of which a subset is associated with decreased gene expression and decreased H3K4me2 levels in mutant embryonic hearts. Our results indicate that KMT2D, through its primary role as an H3K4 di-methyltransferase, is required for regulating specific regulatory programs during heart development.

## RESULTS

### A single copy of *Kmt2d* is sufficient for normal heart development and leads to mild functional defects

**Fig. 1.**
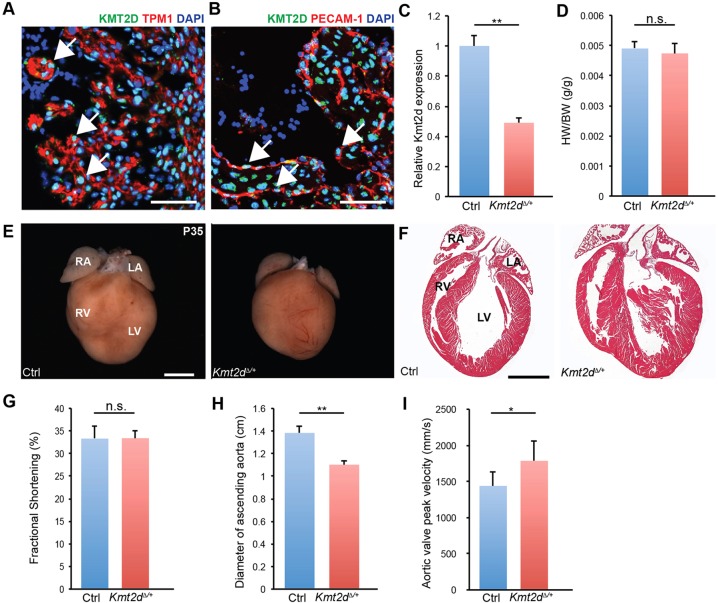
***Kmt2d*^Δ*/+*^ mice have normal cardiac development but exhibit mild narrowing of the ascending aorta.** (A,B) Magnified images of the left ventricle from a four-chamber view section at E12.5 shows (A) KMT2D expression (green) in the nuclei (DAPI, blue) of myocardial cells (TPM1, red) (arrows) and (B) KMT2D expression in the nuclei of endocardial cells (PECAM1, red) (arrows). (C) qRT-PCR for *Kmt2d* transcript levels in E8.0 control and *Kmt2d*^Δ*/+*^ embryos. (D) Heart weight to body weight ratio of P35 control (*n=*6) and *Kmt2d*^Δ*/+*^ (*n=*5) mice. (E) Representative images of P35 control and *Kmt2d*^Δ*/+*^ hearts. (F) Four-chamber view cardiac sections from P35 control and *Kmt2d*^Δ*/+*^ mice stained with Hematoxylin and Eosin (H&E). (G-I) Fractional shortening (G), diameter of the ascending aorta (H) and peak velocity of blood flow through the aortic valve (I) of P35 control and *Kmt2d*^Δ*/+*^ mice. RA, right atrium; LA, left atrium; RV, right ventricle; LV, left ventricle. *P<0.05, **P<0.01; n.s., no significant difference. Error bars indicate s.d. Scale bars: 50 μm in A,B; 2 mm in E; 500 μm in F.

Heart development relies on appropriate gene regulation in multiple cell types. We examined the expression of KMT2D in the developing embryonic mouse heart. Immunofluorescence for KMT2D on embryonic day (E) 9.5 to E12.5 cardiac sections showed ubiquitous expression in the developing heart (Fig. S1A). Closer examination of E12.5 hearts showed expression in both cardiomyocytes (Fig. 1A) and endocardial cells (Fig. 1B).

Kabuki syndrome patients carry truncating mutations in *KMT2D*, which are predicted to result in haploinsufficiency. To determine if *Kmt2d* is haploinsufficient in mice, mice carrying a floxed allele of *Kmt2d* (*Kmt2d^fl^*, referred to as *Mll4^f^* in Lee et al., 2013) were crossed to transgenic *ACTB-Cre* mice (Lewandoski et al., 1997) to generate mice heterozygous for a *Kmt2d*^Δ^ null allele. qRT-PCR confirmed depletion of the *Kmt2d* transcript by 50% in E8.0 *Kmt2d*^Δ*/+*^ embryos compared with control *Kmt2d^+/+^* littermates (Fig. 1C). *Kmt2d*^Δ*/+*^ mice survived to adulthood (Table S1). At postnatal day (P) 35, *Kmt2d*^Δ*/+*^ mice had normal heart weight/body weight (Fig. 1D), and no differences in cardiac morphology compared with littermate wild-type controls (Fig. 1E,F). To determine if *Kmt2d*^Δ*/+*^ mice had altered cardiac function, we performed echocardiography on P35 *Kmt2d*^Δ*/+*^ and wild-type controls. There were no significant changes in fractional shortening in *Kmt2d*^Δ*/+*^ mice (Fig. 1G), but measured a significant narrowing of the diameter of the ascending aorta (Fig. 1H) and increased aortic valve peak velocity (Fig. 1I).

We conclude that a single copy of *Kmt2d* is sufficient for normal mouse heart development and function, with mild defects in the ascending aorta.

### Conditional deletion of *Kmt2d* in cardiac precursors and myocardium disrupts cardiac development

**Fig. 2.**
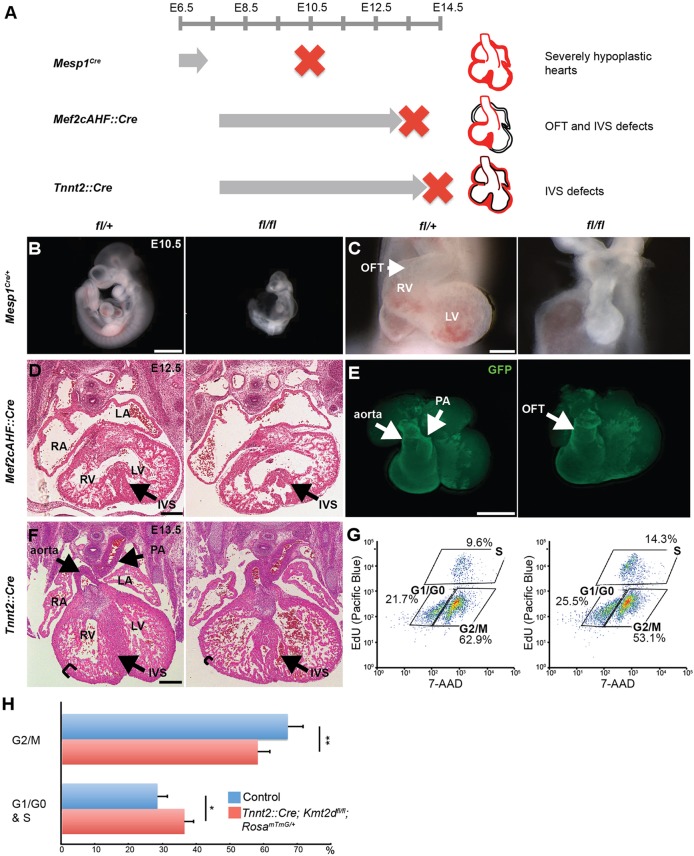
**Deletion of *Kmt2d* in cardiac precursors and myocardium leads to embryonic lethality and cardiac defects.** (A) Schematic overview of *Kmt2d* deletion phenotypes in mesodermal precursors, anterior heart field (AHF) precursors and cardiomyocytes. (B) E10.5 *Mesp1^Cre^;Kmt2d ^fl/fl^* embryos show developmental delay compared with wild-type littermates. (C) E10.5 *Mesp1^Cre^;Kmt2d ^fl/fl^* mutants show severely hypoplastic hearts. (D) E12.5 *Mef2cAHF::Cre;Kmt2d ^fl/fl^* four-chamber view cardiac sections stained with H&E show a disorganized interventricular septum (arrowheads). (E) E12.5 *Mef2cAHF::Cre;Kmt2d ^fl/fl^;Rosa^mTmG/+^* hearts show defects in outflow tract septation (arrowhead). GFP reporter is expressed in Cre-positive cells. (F) E13.5 *Tnnt2::Cre;Kmt2d ^fl/fl^* four-chamber view cardiac sections stained with H&E show disorganized interventricular septum (arrowheads) and thin compact myocardium (brackets) in mutants. (G) E12.5 control (WT) and *Tnnt2::Cre;Kmt2d ^fl/fl^;Rosa^mTmG/+^* (cKO) hearts were labeled with EdU for newly synthesized DNA and EdU-labeled cells were detected with Pacific Blue azide using Click chemistry. Cells were stained with 7-AAD to determine total DNA content and cell cycle distribution was determined by FACS analysis, sorting for Cre-positive cells using GFP reporter. Representative FACS plots of the Cre-positive population show an increase in the number of cells in G1/G0 and S phase and a decrease in G2/M phase in the mutant. (H) Cell cycle analysis of E12.5 control and *Tnnt2::Cre;Kmt2d ^fl/fl^;Rosa^mTmG/+^* hearts (*n=*4 per genotype) shows an 8.0% increase in G1/G0 and S phases (*P*<0.05) and an 8.8% decrease in G2/M phases in mutants (*P*<0.01). RV, right ventricle; LV, left ventricle; PA, pulmonary artery; OFT, outflow tract; IVS, interventricular septum. *P<0.05, **P<0.01. Error bars indicate s.d. Scale bars: 1 mm in B; 200 μm in C; 250 μm in D,F; 500 μm in E.

To determine if KMT2D is required for heart development, we interbred *Kmt2d*^Δ*/+*^ animals to obtain homozygous *Kmt2d*^Δ*/*Δ^ embryos. No live *Kmt2d*^Δ*/*Δ^ offspring were observed (Table S1), and *Kmt2d*^Δ*/*Δ^ embryos at E8.0 lacked somites and headfolds (Fig. S2A). qRT-PCR confirmed depletion of *Kmt2d* in *Kmt2d*^Δ*/*Δ^ mutants (Fig. S2B). Severe morphological defects in the *Kmt2d*^Δ*/*Δ^ embryos indicated an early requirement of *Kmt2d* in embryogenesis, precluding assessment of its role during heart development. Therefore, we used conditional deletion of *Kmt2d* to investigate its role in specific cardiac populations (Fig. 2A).

We deleted *Kmt2d* using *Mesp1^Cre^*, which is expressed in mesodermal precursors (Saga et al., 1999), and *Mef2cAHF::Cre*, which is expressed in anterior heart field (AHF) precursors (Verzi et al., 2005) (Fig. 2A); *Mesp1^Cre^* will delete the ubiquitously expressed *Kmt2d* broadly in E7.0 mesoderm, whereas *Mef2cAHF::Cre* will delete *Kmt2d* in a domain restricted to AHF precursors and pharyngeal arches (Devine et al., 2014; Saga et al., 1999; Verzi et al., 2005). qRT-PCR confirmed a significant decrease in *Kmt2d* transcripts in the hearts of E9.0 mesodermal deletion mutants (*P*<0.05, Fig. S2C) and E11.5 AHF deletion mutants (*P*<0.05, Fig. S2D) compared with heterozygous controls.

To verify that the Cre deletion leads to a loss of KMT2D protein, we used a Cre reporter allele, *Rosa^mTmG^* (Muzumdar et al., 2007). Immunostaining of E10.5 *Mef2cAHF::Cre;Kmt2d^fl/fl^;Rosa^mTmG/+^* hearts showed reduced KMT2D levels in Cre-deleted GFP^+^ cells (Fig. S2E).

No live *Mesp1^Cre^;Kmt2d^fl/fl^* mutants were recovered after E10.5 (Table S2). E10.5 mutants appeared developmentally delayed, exhibiting pericardial edema and a linear heart tube (Fig. 2B,C). Similarly, no live *Mef2cAHF::Cre;Kmt2d^fl/fl^* offspring were observed. *Mef2cAHF::Cre;Kmt2d^fl/fl^* mice were found at Mendelian ratios until E11.5, and this decreased subsequently, with all mutants dying by E13.5 (Table S2). The gross morphology of E12.5 mutant embryos and hearts appeared normal (Fig. S2F,G). However, examination of histological sections of E12.5 mutant hearts revealed a disorganized interventricular septum (Fig. 2D). E12.5 mutant hearts showed a failure of outflow tract septation into the aorta and pulmonary artery (Fig. 2E, Fig. S2H). The cardiac defects of both genotypes indicated that *Kmt2d* is required in cardiac mesoderm and AHF precursors for heart development.

Since mesodermal and AHF precursors contribute to multiple cardiac cell types, we determined if *Kmt2d* was required in cardiomyocytes by deleting *Kmt2d* using *Tnnt2::Cre*, which is expressed in the embryonic myocardium from E7.5 onwards (Jiao et al., 2003). qRT-PCR confirmed a significant decrease of *Kmt2d* transcript in E11.5 mutant hearts, although the *Kmt2d* transcript was not completely lost, which was likely to be due to the contribution of non-myocyte populations such as endocardium and cardiac fibroblasts (*P*<0.05, Fig. S2I). *Tnnt2::Cre;Kmt2d^fl/fl^* embryos were found at Mendelian ratios until E13.5, but no live mutants were observed after E14.5 (Table S2). The gross morphology of E13.5 mutant embryos and hearts appeared normal (Fig. S2J,K). Examination of histological sections of E13.5 mutant hearts revealed thin compact myocardium and disorganized ventricular septum (Fig. 2F), similar to the AHF precursor deletion phenotype. However, myocardial deletion mutants showed normal septation of the outflow tract into the aorta and pulmonary artery.

Hypoplasia of the compact myocardium in *Tnnt2::Cre;Kmt2d^fl/fl^* mutants suggested decreased cardiomyocyte proliferation. To test this hypothesis, we performed cell cycle analysis on E12.5 control and mutant hearts (*n=*4 for each genotype). Cell cycle distribution was determined by EdU and 7-AAD double staining, sorting for Cre-positive cells with GFP fluorescence (Fig. 2G). In controls, 66.9% of the Cre-positive cell population was in the G2/M phase, which decreased to 58.0% in the mutants. In controls, 28.3% of the Cre-positive cell population was in the G1/G0 and S phases, which increased to 36.3% in the mutants. The cell population in the G1/G0 and S phases also increased from 28.3% to 36.3% (Fig. 2H). This indicated that a portion of cells is arrested in the G1/G0 and S phases, which could contribute to hypoplasia of the compact myocardium.

These results indicate that *Kmt2d* is required in cardiac precursors and myocardium during heart development, with distinct phenotypes suggesting that *Kmt2d* plays specific roles in each cardiac population.

### Loss of *Kmt2d* leads to downregulation of ion transport genes and altered calcium handling in ventricular myocytes

To assess the transcriptional consequences of *Kmt2d* loss during heart development, we performed global gene expression analyses of embryonic hearts. To avoid confounding secondary effects, we obtained embryonic hearts at the developmental stages when the earliest cardiac defects were observed. For *Mesp1^Cre^* crosses, we used E9.0 hearts (*n*=4 per genotype). For *Mef2cAHF::Cre*, we used E11.5 right ventricles and outflow tracts (*n*=3 per genotype), which are the regions where *Kmt2d* is deleted. For *Tnnt2::Cre* crosses, we used E11.5 embryonic hearts (*n*=3 per genotype).

**Fig. 3.**
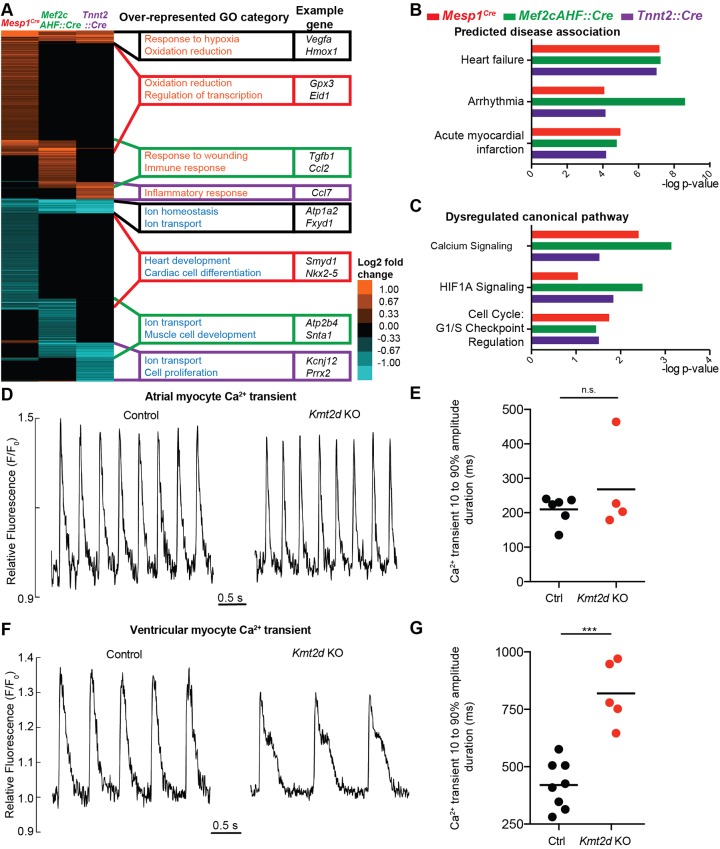
**Deletion of *Kmt2d* in cardiac precursors and myocardium leads to downregulation of ion transport genes and altered calcium handling in ventricular cardiomyocytes.** (A) RNA-Seq analysis comparing differentially expressed genes in E9.0 *Mesp1^Cre^;Kmt2d ^fl/fl^* mutant hearts, E11.5 *Mef2cAHF::Cre;Kmt2d ^fl/fl^* right ventricles and outflow tract and E11.5 *Tnnt2::Cre;Kmt2d ^fl/fl^* mutant hearts (FDR<0.05). (B) IPA of differentially expressed genes in all three deletion genotypes shows that common disease associations that were significantly predicted are related to heart failure (*P*<0.05). (C) IPA shows that common canonical pathways that were significantly dysregulated are associated with calcium signaling, HIF1A signaling and G1/S cell cycle checkpoint regulation (*P*<0.05). (D) Representative Fluo-4 fluorescence recordings from control and *Tnnt2::Cre;Kmt2d ^fl/fl^* (*Kmt2d* KO) atrial myocytes isolated at E11.5. (E) Mean durations of Ca^2+^-dependent Fluo-4 fluorescence transients plotted for control and *Kmt2d* KO atrial myocytes. Each point represents the Ca^2+^ transient duration from myocytes representing one embryonic heart, as determined at the level between 10% of the upstroke and 90% of the decay. An average of 5.8 samples (cells or clusters) were combined per point. n.s., no significant difference. (F) Representative Fluo-4 fluorescence recordings (upper panel) from control and *Kmt2d* KO ventricular myocytes isolated at E11.5. Typically, the Ca^2+^ transients, expressed relative to diastolic fluorescence (F_0_), showed similar peak amplitudes but strong differences in duration due to the presence of a late shoulder or plateau in the *Kmt2d* KO myocytes. (G) Mean durations of Ca^2+^-dependent Fluo-4 fluorescence transients plotted for control and *Kmt2d* KO ventricular myocytes. An average of 23 samples (cells or clusters) were combined per point. *Kmt2d* KO ventricular myocytes had a significantly prolonged duration at 819±137 ms (*n=*5) compared with controls at 420±103 ms (*n=*8) (***P<0.001).

At a false discovery rate (FDR) <0.05, we found 2226 genes dysregulated in *Mesp1^Cre^* mutants, 1212 genes dysregulated in *Mef2cAHF::Cre* mutants and 774 genes dysregulated in *Tnnt2::Cre* mutants (Fig. 3A, Table S3). Average-linkage cluster analysis showed that differentially expressed genes in *Mesp1^Cre^*, *Mef2cAHF::Cre* and *Tnnt2::Cre* mutants mostly clustered separately, with some overlap (Fig. 3A), indicating that *Kmt2d* regulates distinct subsets of genes in each cardiac population. Gene ontology (GO) analysis revealed that the distinct subsets of genes upregulated in the three deletion mutants were enriched for functions in hypoxia response, whereas downregulated genes were enriched for functions in ion transport and homeostasis (Fig. 3A). Downregulation of genes related to ion transport, such as *Atp1a2*, *Snta1*, *Camk2a* and *Fxyd1*, were validated by qRT-PCR in *Tnnt2::Cre;Kmt2d^flfl^* deletion mutants (Fig. S3A).

To determine biological functions commonly dysregulated in all three cardiac deletion mutants, we analyzed this dataset using Ingenuity pathway analysis (IPA). Predicted disease associations included heart failure and myocardial infarction (Fig. 3B), suggesting strong disruption of cardiac function and circulatory failure. We observed that common dysregulated canonical pathways included calcium signaling, hypoxia signaling and G1/S cell cycle checkpoint regulation (Fig. 3C). In addition, common dysregulated biological functions included anemia, muscle contractility, ion homeostasis and reactive oxygen species (ROS) (Fig. S3B). We also observed that the predicted dysregulation of upstream regulators included multiple hypoxia response genes, such as *Hif1a* and *Commd1* (Fig. S3C). We analyzed the *Tnnt2::Cre* deletion gene expression dataset further using an unbiased gene set enrichment analysis (GSEA) (Subramanian et al., 2005). Similarly, we observed a significant decrease in inorganic anion transport genes and erythropoietic markers, as well as an enrichment of hypoxia response genes (FDR<0.01, Fig. S3D-F).

To determine if the decrease in ion transport gene expression led to a loss of protein expression, we examined the expression of ATP1A2, the alpha-2 isoform of the Na^+^,K^+^-ATPase, in *Tnnt2::Cre;Kmt2d^fl/fl^* and control hearts. In the left atria and interventricular septum, ATP1A2 was decreased in Cre-positive mutant cardiomyocytes (Fig. S4A,B). By contrast, HIF1A, a key hypoxia response factor, was detected predominantly in Cre-negative cells (Fig. S4C), suggesting that the increase in hypoxic response gene expression might be a secondary response.

In the myocardial deletion mutants, downregulated ion transport genes included *Snta1* and *Fxyd1.* SNTA1 associates with the cardiac sodium channel SCN5A and the plasma membrane Ca^2+^-ATPase PMCA4B (also known as ATP2B4), and mutations in *SNTA1* are associated with long QT syndrome 12 (Ueda et al., 2008). *Fxyd1* encodes a sarcolemmal protein (also known as phospholemman) that regulates the ion channels Na^+^,K^+^-ATPase and sodium-calcium exchanger NCX1 (also known as SLC8A1), and thus exerts effects on intracellular Ca^2+^ concentration. *Fxyd1-*deficient cardiomyocytes have increased Na^+^/Ca^2+^ exchange current (Zhang et al., 2006), which is associated with increased action potential (AP) duration. These studies suggest downregulation of *Snta1* and *Fxyd1* might lead to altered intracellular calcium levels ([Ca^2+^]_i_) in cardiomyocytes.

Accordingly, we measured spontaneous [Ca^2+^]_i_ transients in E11.5 atrial and ventricular myocytes from mutants and controls to ascertain whether myocardial deletion of *Kmt2d* leads to altered Ca^2+^ handling. In atrial myocytes, mutant [Ca^2+^]_i_ transient waveforms were similar to those of controls (Fig. 3D) and the mean duration, measured at the level delimited by 10% of the initial rise and 90% of the final decay, showed no significant differences between the genotypes (Fig. 3E). However, in the ventricular myocytes, mutant [Ca^2+^]_i_ transients exhibited prolonged waveforms (Fig. 3F), reflecting a prolonged AP duration (Fig. S4D). The mean duration was 819±137 ms (*n*=5) in mutants, approximately double that of controls (420±103 ms, *n*=8) (*P*<0.001, Fig. 3G), indicating a specific role for *Kmt2d* in regulating calcium handling in the ventricular myocardium. Because we performed RNA-Seq on whole hearts, we were not able to discern which dysregulated ventricular genes were primarily responsible for the electrophysiological abnormalities.

We conclude that *Kmt2d* regulates distinct subsets of genes in mesodermal precursors, AHF precursors and cardiomyocytes, but also generally controls the ion transport gene expression response. Reduced ion transport gene expression is likely to lead to prolonged Ca^2+^ transient duration in mutant ventricular myocytes, which reflects prolonged AP duration that might predispose mutants to arrhythmias.

### Myocardial deletion of *Kmt2d* results in decreased H3K4me1 and H3K4me2 at enhancers and promoters

Since *Kmt2d* encodes an H3K4 methyltransferase, we sought to determine if myocardial deletion of *Kmt2d* leads to changes in H3K4 methylation levels. Western blot analysis did not show any decrease in the bulk levels of H3K4me1, H3K4me2 or H3K4me3 (Fig. S5A). To determine if there was a decrease in H3K4 methylation at specific genomic loci, we performed chromatin immunoprecipitation coupled with sequencing (ChIP-Seq) for H3K4me1, H3K4me2 and H3K4me3 on E11.5 control and mutant hearts (*n*=90 for each genotype). Hearts from each genotype were pooled to give three biological replicates, and we analyzed the data in conjunction with H3K27Ac ChIP-Seq data from wild-type E11.5 hearts (Nord et al., 2013). We then examined the enrichment of the histone methylation marks at the transcription start site (TSS) and non-TSS H3K27Ac-enriched sites, which marks active enhancers.

**Fig. 4.**
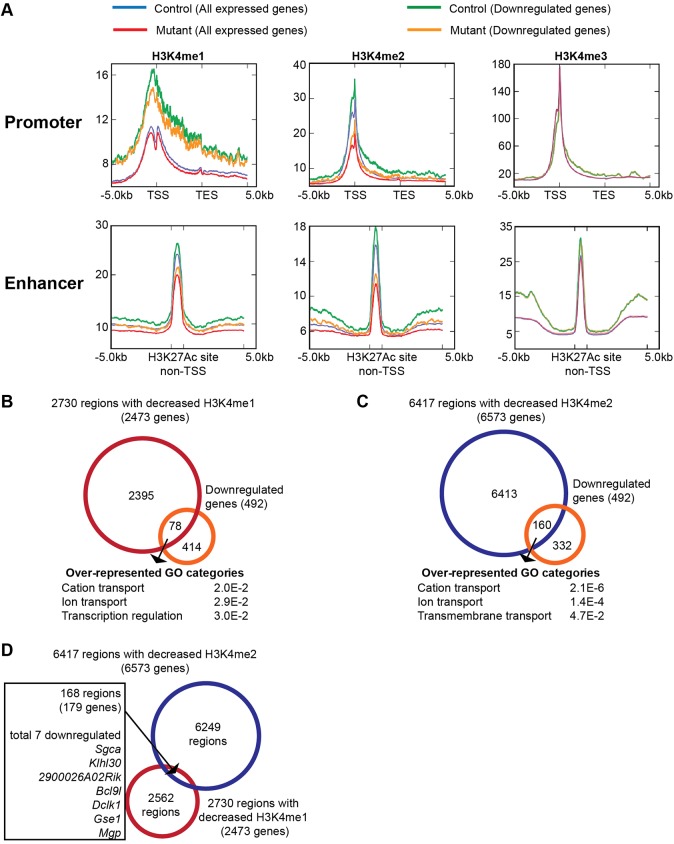
**Myocardial deletion of *Kmt2d* results in a decrease in average H3K4me1 and H3K4me2 levels at enhancers and promoters.** (A) Metagene profiles showing the average distribution of H3K4me1, H3K4me2 and H3K4me3 input-normalized tag density at promoters and enhancers in E11.5 control and *Tnnt2::Cre;Kmt2d ^fl/fl^* hearts. For all expressed genes and 492 downregulated genes analyzed, mutants show decreased H3K4me1 levels at enhancers, decreased H3K4me2 levels at promoters and enhancers, and no difference in H3K4me3 levels. (B) 2730 regions with decreased H3K4me1 levels (FDR<0.1) are assigned to 2473 genes by proximity using Stanford GREAT. GO categories of 78 downregulated genes with decreased H3K4me1 in mutant hearts are over-represented for ion transport genes. (C) 6417 regions with decreased H3K4me2 levels (FDR<0.1) are assigned to 6573 genes by proximity using GREAT. GO categories of 162 downregulated genes with decreased H3K4me2 are over-represented for ion transport genes. (D) Venn diagram representing the overlap of 2730 regions with decreased H3K4me1 with 6417 regions with decreased H3K4me2. Of the overlapping 168 regions mapped to 179 genes, only seven genes are downregulated in E11.5 *Tnnt2::Cre;Kmt2d ^fl/fl^* hearts. TSS, transcription start site; TES, transcription end site.

Average Metagene profile plots revealed a statistically significant decrease in H3K4me2 levels at the TSS and at non-TSS H3K27Ac-enriched sites in the *Tnnt2::Cre;Kmt2d ^fl/fl^* mutant (Fig. 4A, blue and red lines). However, there were no significant changes in the levels of H3K4me1 and H3K4me3 at the TSS. Similarly, at non-TSS H3K27Ac-enriched sites, there were no changes in H3K4me3 levels, although there was a modest decrease in H3K4me1 levels.

We further examined average profile plots for the 492 genes that were downregulated in the myocardial deletion mutants. We observed a marked decrease in mutant H3K4me2 at the TSS and non-TSS H3K27Ac-enriched sites, with less noticeable differences in H3K4me1 and H3K4me3 (Fig. 4A, green and orange lines). This is similar to the global pattern, although this subset of 492 genes has higher average levels of H3K4me1 and H3K4me2 at both the TSS and non-TSS H3K27Ac-enriched sites compared with the global average.

We called 358,833 total merged peaks across all replicates of H3K4me1, H3K4me2 and H3K4me3. From these regions, we identified 2730 (0.8%) with decreased H3K4me1 (FDR<0.1) and 6417 (1.8%) with decreased H3K4me2 (FDR<0.1, Table S3), mapping to 2473 and 6573 genes, respectively. This indicates that only a subset of genomic loci had decreased H3K4me1 and H3K4me2. Most of the regions with decreased H3K4me1 were located at distal regulatory elements (Fig. S5B), similar to the global H3K4me1 enrichment (Table S4). By contrast, 54.2% of decreased H3K4me2 region-gene associations were proximal to a TSS, whereas 45.8% were located distal to a TSS (Fig. S5C). This indicates an approximately equal distribution of regions with decreased H3K4me2 at proximal and distal regulatory elements, whereas the global H3K4me2 enrichment is mostly at distal regulatory elements (88.7%, Table S4).

Of 492 genes downregulated in myocardial deletion mutants, 78 (15.9%) had decreased H3K4me1 levels (Fig. 4B) and 162 (32.9%) had decreased H3K4me2 levels (Fig. 4C), with enrichment of ion transport-related genes in these subsets (*P*<0.05). Compared with all downregulated genes, this enrichment in ion transport function is greater than expected for downregulated genes with decreased H3K4me2 (*P=*0.025), but not decreased H3K4me1 (*P=*0.22) (Table S5). The majority of genes with decreased H3K4me1 (2395 genes, 96.8%) and decreased H3K4me2 (6413 genes, 97.6%) did not show changes in gene expression, suggesting that a decrease in H3K4me1 or H3K4me2 levels is not sufficient for a decrease in gene expression and might require crosstalk with other mechanisms regulating transcription. It is possible that decreased H3K4me1 and H3K4me2 levels may be due to indirect effects. However, we might also fail to detect many regions with decreased H3K4 methylation levels in cardiomyocytes by examining embryonic hearts with heterogeneous cell populations. Nonetheless, we find that, compared with all expressed genes with decreased H3K4 methylation, downregulated genes are significantly more likely to be associated with decreased H3K4me1 regions (*P*=0.025) and decreased H3K4me2 regions (*P*=0.054) (Table S6).

Interestingly, only 168 regions have reductions in both H3K4me1 and H3K4me2 levels, corresponding to 6.2% of regions with decreased H3K4me1 or 2.6% of regions with decreased H3K4me2 (Fig. 4D). Of these 168 regions, only seven mapped to downregulated genes, suggesting that KMT2D has distinct roles in maintaining H3K4me1 and H3K4me2 levels at different genomic regions. No decrease in H3K4me1 and H3K4me3 levels was observed at the TSS of *E2f2* despite a substantial decrease in H3K4me2 levels (Fig. S5D), further illustrating that KMT2D is highly specific in regulating H3K4me1 and H3K4me2 levels at these genomic loci in the embryonic heart.

### KMT2D binds to genomic regions associated with cell cycle, hypoxia-reoxygenation and ion transport genes in cardiomyocytes

To determine if KMT2D localizes to genomic regions to directly maintain H3K4me2 levels, we performed ChIP-Exo for KMT2D. Since E11.5 hearts had limited cell numbers, we used cardiomyocytes derived from embryonic stem cells (ESCs) (Wamstad et al., 2012). To validate the use of ESC-derived cardiomyocytes, we compared H3K4me1 and H3K4me3 enrichment in E11.5 hearts, ESC-derived cardiomyocytes, and ESCs; this demonstrated strong correlations in H3K4 methylation profiles between E11.5 hearts and differentiated cardiomyocytes, distinct from the ESC profiles (Fig. S6A).

**Fig. 5.**
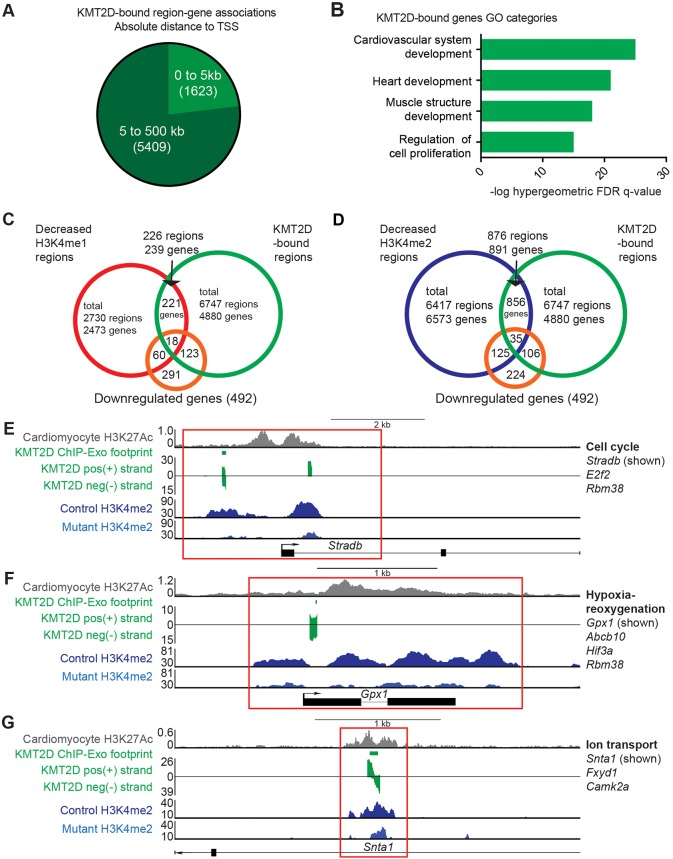
**KMT2D binds to promoter and enhancer regions of genes related to cell cycle, hypoxia-reoxygenation and ion transport.** (A) 6747 regions bound by KMT2D in *in vitro* cardiomyocytes are assigned to 4880 genes by proximity using Stanford GREAT. 1623 region-gene associations are within 5 kb of a TSS and 5409 region-gene associations are 5 to 100 kb of a TSS. (B) GO categories of KMT2D-bound genes are over-represented for heart development and cell proliferation. (C) Venn diagram showing that only a subset of 18 genes overlap between 492 downregulated genes in E11.5 *Tnnt2::Cre;Kmt2d^fl/fl^* hearts and regions bound by KMT2D with decreased H3K4me1 levels (FDR<0.1). (D) Venn diagram showing that only a subset of 35 genes overlap between 492 downregulated genes in E11.5 *Tnnt2::Cre;Kmt2d^fl/fl^* hearts and regions bound by KMT2D with decreased H3K4me2 levels (FDR<0.1). (E) Representative browser tracks of KMT2D ChIP-Exo positive strand, negative strand, and resulting footprint shows that KMT2D binds to the TSS of the cell cycle gene *Stradb* in cardiomyocytes, which corresponds to a region with decreased H3K4me2 (FDR<0.1) in mutant hearts (red box). (F) KMT2D binds to the TSS of the antioxidant enzyme gene *Gpx1* in cardiomyocytes, which corresponds to a region with decreased H3K4me2 (FDR<0.1) in mutant hearts (red box). (G) KMT2D binds to the intronic H3K27Ac-enriched enhancer of the ion transport gene *Snta1* in *in vitro* cardiomyocytes, which corresponds to a region with decreased H3K4me2 (FDR<0.1) in mutant hearts (red box). The H3K27Ac browser track *y*-axis corresponds to reads per million; for the other tracks the *y*-axis corresponds to input-normalized tag density.

We identified 6747 genomic regions bound by KMT2D (Table S3), which mapped to 4880 genes. 23.1% of region-gene associations were located within 5 kb of the TSS, whereas 76.9% were located 5 to 100 kb from the TSS (Fig. 5A). Thus, most KMT2D binding localizes to distal regulatory regions.

We examined KMT2D-bound regions and found that GO categories such as heart development and cell proliferation were over-represented (Fig. 5B). Motif analysis of the KMT2D-bound regions revealed enrichment of motifs matching transcription factors with crucial functions in cardiac development (Fig. S6B), including HIF1A, MYC, SP3, TEAD1 and SRF (Iyer et al., 1998; Moens et al., 1993; van Loo et al., 2007; He et al., 2011; Parlakian et al., 2004). This suggests that the KMT2D methyltransferase complex may be recruited by these transcription factors. We further examined these KMT2D-bound regions for changes in H3K4 methylation in E11.5 hearts; although this comparison is not ideal owing to the different cellular origins and the heterogeneity of the E11.5 heart, the comparison yielded significant results. Similar to the trend observed in all expressed genes, we observed a greater decrease in H3K4me2 levels than H3K4me1 levels, and no change in H3K4me3 levels (Fig. S6C), suggesting that KMT2D is required at specific genomic loci to maintain H3K4me1 and H3K4me2 levels.

Finally, we investigated which KMT2D direct targets require KMT2D to maintain H3K4me2 levels and gene expression. We found that a small percentage of KMT2D-bound regions (3.4%, 226 regions corresponding to 239 genes) exhibited decreased H3K4me1 levels in E11.5 myocardial deletion mutant hearts, and a subset of 18 genes showed a concomitant decrease in gene expression (Fig. 5C; Table S7). There were no enriched functions for this subset of 18 genes. Since E11.5 hearts have a heterogeneous cell population including non-myocytes, the overlap between KMT2D-bound genes and genes with decreased H3K4 methylation levels might be under-represented. We have further examined KMT2D-bound regions with decreased H3K4me2 (13%, 876 regions corresponding to 891 genes) and found greater overlap with downregulated genes (35 genes, Fig. 5D; Table S7).

In this subset of 35 genes, which represents KMT2D direct targets in cardiomyocytes that require KMT2D for normal H3K4me2 levels and gene expression in E11.5 hearts, we identified genes related to cell cycle, hypoxia-reoxygenation and ion transport. This includes *Stradb* (Fig. 5E), which encodes a pseudokinase implicated in G1 phase cell cycle arrest (Boudeau et al., 2006), and *Gpx1* (Fig. 5F), which encodes an antioxidant enzyme that protects cardiac mitochondria against reoxygenation-induced ROS (Thu et al., 2010). The subset also included the ion transport genes *Snta1* and *Fxyd1* (Fig. 5G), which suggests that the electrophysiological defects observed in *Kmt2d*-deficient cardiomyocytes might be due to direct KMT2D regulation of ion transport gene expression via H3K4 di-methylation.

## DISCUSSION

**Fig. 6.**
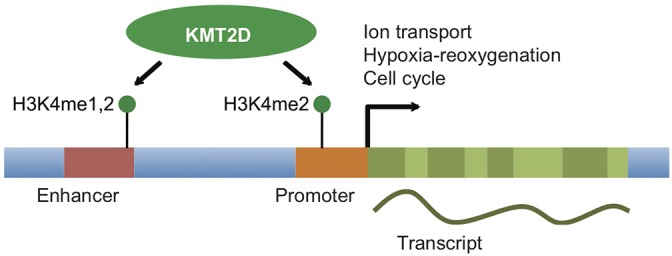
**KMT2D is required for H3K4 di-methylation (and mono-methylation) to maintain specific gene expression programs in heart development.** Diagrammatic representation of a protein-coding gene, including promoter and enhancer, acted upon by KMT2D to deposit the histone modifications H3K4me1 and H3K4me3.

We identified an essential role for KMT2D as a regulator of heart development. Our work demonstrates that *Kmt2d* is required in cardiac precursors and cardiomyocytes during cardiogenesis, with distinct phenotypes and dysregulated genes that suggest *Kmt2d* plays specific roles in each cardiac population. In the absence of *Kmt2d*, ion transport genes are downregulated in embryonic hearts, with a corresponding cellular phenotype with altered calcium handling. We further show that KMT2D is required for H3K4 mono- and di-methylation at promoters and enhancers of a subset of genes. In particular, we identified the requirement for KMT2D for H3K4 di-methylation at several genes related to ion transport, hypoxia-reoxygenation and cell cycle. Taken together, our findings suggest that KMT2D acts primarily as an H3K4 di-methyltransferase to regulate a specific cardiac transcriptional program for ion homeostasis and heart development (Fig. 6).

### KMT2D is required for H3K4 mono- and di-methylation at enhancers and promoters

Most studies have focused on the association of H3K4me1 with enhancers and H3K4me3 with promoters of actively transcribed genes, but less is understood about the dynamics of H3K4me2 distribution and its contribution to gene expression (Ruthenburg et al., 2007; Shilatifard, 2012). Previous work identified H3K4me2 enrichment at enhancers, defining lineage-specific recruitment sites on chromatin (Lupien et al., 2008; Ong and Corces, 2011). H3K4me2 is also enriched in genomic regions surrounding the TSS of tissue-specific genes, with dynamic distribution during cell differentiation and development, suggesting that H3K4me2 enrichment at promoter regions plays a key role in regulating tissue-specific gene expression (Pekowska et al., 2010; Zhang et al., 2012; Popova et al., 2012).

Studies in mammalian cells have uncovered the role of KMT2D as an H3K4 mono- and di-methyltransferase at enhancer or promoter regions (Hu et al., 2013; Guo et al., 2013; Lee et al., 2013; Kaikkonen et al., 2013; Ortega-Molina et al., 2015; Zhang et al., 2015). In agreement with these studies, we identified KMT2D-bound regions at both promoters and enhancers of cardiac genes. In the absence of *Kmt2d*, we observe that a subset of these genes is downregulated, with a corresponding decrease in H3K4me2 levels at enhancers or promoters and gene bodies at KMT2D-bound regions. We also observe a decrease in H3K4me1 levels at a smaller subset of enhancer regions, which are distinct from regions with decreased H3K4me2 levels. Our results suggest that KMT2D is an H3K4 mono- and di-methyltransferase required both at enhancers and promoters for active gene expression, with a specific role for KMT2D as an H3K4 di-methyltransferase at promoters. It will be important in future studies to gain a better understanding of the significance of specific H3K4 di-methylation distribution at promoters or enhancers for transcription activation.

Although we observed several thousand genomic regions with decreased H3K4me2 or H3K4me1, only a small subset of genes within these groups was downregulated. This suggests that not all genes are sensitive to a loss in H3K4me1 or H3K4me2 levels. There might be a temporal delay in H3K4me1- or H3K4me2-dependent gene expression, or some genes are downregulated at a later developmental stage. Alternately, changes in H3K4me1 or H3K4me2 levels alone might not effect a direct change in gene expression. Further studies of genetic interactions between *Kmt2d* and other histone modifiers might provide additional insight into histone crosstalk and the epigenetic regulation of gene expression.

### KMT2D regulates cardiac gene expression related to ion transport, hypoxia-reoxygenation and the cell cycle

We found that myocardial deletion of *Kmt2d* leads to changes in ion transport gene expression and altered calcium handling in ventricular myocytes, indicating a role for KMT2D in regulating ion homeostasis. A similar observation has been made for adult cardiomyocyte deletion of *Paxip1*, which encodes a KMT2D complex-specific subunit, leading to changes in ion channel gene expression and arrhythmias (Stein et al., 2011). Furthermore, a child with severe Kabuki syndrome was reported to have bradycardia and asystole (Shah et al., 2005). These data suggest that the KMT2D complex has an important role in regulating cardiac electrophysiology.

Functional ion channel activity is crucial in the embryonic heart, particularly during mid-gestation. In rats treated during gestation, teratogenic doses of potassium channel blockers increased embryonic death associated with a significantly increased incidence of cardiac defects, particularly ventricular septal defects and great vessel abnormalities, similar to what we observed in *Kmt2d* mutants (Webster et al., 1996; Abela et al., 2010). The channel-blocking drugs induce cardiac arrhythmia and heart failure in the embryos, leading to chronic hypoxia-reoxygenation damage, thus adversely affecting cardiogenesis and resulting in cardiac malformations (Wellfelt et al., 1999; Danielsson et al., 2001, 2007; Sköld et al., 2001). Our results are consistent with this observation, as *Kmt2d* mutants exhibit downregulation of ion transport genes and concomitant upregulation of hypoxia response genes. This suggests that disruption of ion channel activity due to *Kmt2d* deletion could contribute to the cardiac defects observed in mutants.

We also identified potential KMT2D target genes that might protect the heart from hypoxia-reoxygenation damage. GPX1 and ABCB10 protect cardiac mitochondria against hypoxia-reoxygenation-induced oxidative stress, whereas HIF3A and RBM38 are negative regulators of HIF1A (Thu et al., 2010; Bayeva et al., 2013; Forristal et al., 2010; Cho et al., 2015). Cardiac mitochondria respond to hypoxia by increasing the generation of ROS, subjecting the heart to oxidative stress (Duranteau et al., 1998). This suggests that decreased expression of these genes could exacerbate hypoxia-reoxygenation injury in mutant hearts. *Stradb*, *E2f2* and *Rbm38* are important for cell cycle decisions between G0/G1 and S phase (Boudeau et al., 2006; Infante et al., 2008; Miyamoto et al., 2009), suggesting that the cell cycle defects that we observed in myocardial deletion mutants could be due to dysregulation of these genes.

It is likely that the mutant cardiac phenotypes are the combined result of multiple dysregulated KMT2D target genes. To better understand how KMT2D regulates heart development, it will be important to examine how KMT2D target genes interact during cardiogenesis and to determine the cardiac transcription factors that recruit the KMT2D complex for cell type-specific gene expression. Although Kabuki syndrome is a haploinsufficient, multi-organ syndrome in humans, our studies in cardiac-specific knockouts shed light on the primary nature of cardiac defects in the absence of KMT2D and uncover possible etiologies for CHDs in Kabuki syndrome patients.

In conclusion, we have discovered important and novel roles for *Kmt2d* in H3K4 mono- and di-methylation during heart development. Our work adds to the understanding of the transcriptional regulation of cardiac gene expression during heart development and highlights the contribution of histone modifiers. In future work, it will be important to understand how the distribution and dynamics of H3K4 mono- and di-methylation might interact with other transcriptional signals to drive transcription activation. It will also be important to establish whether KMT2D regulates ion homeostasis in other organ systems, and thus identify potential therapies for the multiple congenital anomalies observed in Kabuki syndrome.

## MATERIALS AND METHODS

### Mice

*Kmt2d^fl/fl^* [referred to as *Mll4^f/f^* in Lee et al. (2013)], *ACTB::Cre* (Lewandoski et al., 1997), *Mesp1^Cre^* (Saga et al., 1999), *Mef2cAHF::Cre* (Verzi et al., 2005), *Rosa^mTmG^* (Muzumdar et al., 2007), *Tnnt2::Cre* (Jiao et al., 2003) and *Tie2::Cre* (Kisanuki et al., 2001) mice have been described previously. These mice were backcrossed for at least five generations to a C57BL/6 background. Animals were treated in accordance with the guidelines of the University of California, San Francisco (UCSF) Institutional Animal Care and Use Committee (IACUC). Conceptuses were generated from timed matings and detection of the vaginal plug was considered as E0.5.

### Echocardiography

Echocardiograms to assess systolic function were performed using the Vevo 770 High-Resolution Micro-Imaging System (VisualSonics). M-mode and two-dimensional measurements were made as described previously (Li et al., 2012). The measurements were made from five *Kmt2d*^Δ*/+*^ heterozygous mutants (*n*=5) and six wild-type control littermates (*n*=6).

### Immunofluorescence and western blotting

Embryonic trunk regions were dissected out and fixed in 4% paraformaldehyde for 30 min, followed by serial incubations in 10%, 20% and 30% sucrose, then frozen in Tissue-Tek OCT Compound (Sakura Finetek). Cryosections (8 μm) were mounted on glass slides, dried for 20 min at room temperature, washed with PBS containing 0.1% Tween-20 (PBST) and incubated in blocking buffer (0.1 M Tris HCl pH 7.5, 0.15 M NaCl, 0.5% blocking reagent; PerkinElmer) for 1 h. Samples were incubated with primary antibodies at 4°C overnight in blocking buffer, washed with PBST and incubated with secondary antibodies for 1 h. Slides were then washed, stained with 1 μg/μl DAPI and mounted in Prolong Gold Antifade (Life Technologies). Whole-mount immunostaining of E12.5 hearts is described in the supplementary Materials and Methods. Western blotting was performed using standard techniques. Antibodies are listed in Table S8.

### Cell cycle analysis

200 μl 5 mg/ml 5-ethnyl-2′-deoxyuridine (EdU) in PBS was injected per 8-week-old female mouse. After 2 h, E12.5 whole hearts were dissected and dissociated into single cells by treatment with TrypLE Express (Life Technologies) for 3 min at 37°C. Dissociated single cells were fixed and EdU detection was performed using the Click-iT Pacific Blue Flow Cytometry Assay Kit (Life Technologies). Cells were stained with 7-aminoactinomycin D (7-AAD; Life Technologies) for 30 min and analyzed on an LSR II flow cytometer (BD Biosciences). Data were analyzed using FlowJo software.

### RNA isolation and quantitative PCR

Total RNA was isolated from embryonic hearts using the RNAqueous Micro Total RNA Isolation Kit (Ambion/Life Technologies). cDNA was generated using the High Capacity cDNA Reverse Transcriptase Kit (Applied Biosystems/Life Technologies) and qRT-PCR reactions were performed in triplicate using the Power SYBR Green Master Mix (Applied Biosystems) and run on a 7900HT Real-Time PCR system (Applied Biosystems). Relative abundance of mRNAs was calculated by normalization to *Actb* mRNA levels. Quantitative PCR data in all figures are presented as mean±s.d. Primer sequences are listed in Table S9.

### RNA-Seq analysis

Whole-genome gene expression analysis was performed on RNA isolated from control and mutant embryonic hearts. Libraries were prepared using Illumina TruSeq Paired-End Cluster Kit v3, and sequenced with the Illumina HiSeq 2500 system for pair-ended 100 bp reads. Reads were aligned to the reference assembly NCBI37/mm9 (mouse) and assigned to genes using FeatureCounts (Liao et al., 2014). Differential expression was calculated using USeq (Nix et al., 2008). Differentially expressed genes were filtered with thresholds of FDR<0.05, clustered using Cluster 3.0 and visualized with Treeview (Eisen et al., 1998). GO analysis was conducted using DAVID (Huang et al., 2009). RNA-Seq datasets were analyzed and functional analyses were generated through the use of IPA (Qiagen, www.qiagen.com/ingenuity). GSEA was performed as described (Subramanian et al., 2005). RNA-Seq data have been deposited at GEO under accession numbers GSE74679 and GSE75151.

### Calcium transients and electrophysiology

Cardiomyocytes isolated from E11.5 embryos were plated onto laminin-coated coverslips (#1, Warner Instruments). Briefly, beating myocytes were loaded with Fluo-4 (Thermo Fisher) for 20 min and then de-esterified for 20 min. Ca^2+^-sensitive fluorescence signals were recorded using a filter set centered at 480 nm excitation and 535 nm emission (#49011, Chroma Technology), then low-pass filtered at 2 kHz and digitized at 5 kHz for 30 s per data file. Mean background emission was subtracted and changes in fluorescence amplitudes were expressed relative to the mean diastolic level attained between spontaneous beats (i.e. F/F_o_). In a small number of additional experiments, Fluo-4-loaded cells were subjected to amphotericin B-perforated patch clamp (Spencer et al., 2014). Detailed methods are provided in the supplementary Materials and Methods.

### Chromatin immunoprecipitation (ChIP)

Ninety E11.5 *Tnnt2::Cre;Kmt2d^fl/fl^* hearts and 90 *Tnnt2::Cre;Kmt2d^fl/+^* control hearts were crosslinked with 1% formaldehyde, quenched with 125 mM glycine, washed in PBS, resuspended in lysis buffer and sonicated using a Bioruptor Standard (Diagenode). ChIP of histone modifications (ChIP-Seq) was performed according to Alexander et al. (2015) with minor modifications. For each antibody (H3K4me1, H3K4me2 and H3K4me3; Table S8) 3 μg were added to three control samples and three mutant samples (three biological replicates per genotype per antibody).

For each ChIP-Exo replicate, 5×10^7^ cardiac myocytes were derived from E14 mouse ESCs via directed differentiation as previously described (Wamstad et al., 2012). Samples were incubated with 2 mM disuccinimidyl glutarate for 45 min before the crosslinking step. ChIP-Exo was performed as previously described (Serandour et al., 2013; Alexander et al., 2015) using 8 μg anti-KMT2D antibody (Lee et al., 2013). DNA libraries were gel purified and sequenced on an Illumina HiSeq 4000 to generate 50 bp single-end reads with >100 million reads per sample. The ChIP-Seq and ChIP-Exo analysis pipeline is provided in the supplementary Materials and Methods. RNA-Seq data have been deposited at Gene Expression Omnibus (GEO) under accession number GSE74679. ChIP-Seq and ChIP-Exo data have been deposited at GEO under accession number GSE75151.

### Statistical analyses

Data are reported as mean±s.d. and calculated using GraphPad Prism 6 software. Student's *t*-test (unpaired, two-tailed) was used when comparing mutant and control groups, with *P*<0.05 considered significant. Hypergeometric tests were performed to determine whether genes with decreased expression were more likely to be correlated with genes with decreased H3K4 methylation. The expressed gene set contained any gene with FPKM≥0.1 in either the control or *Tnnt2::Cre;Kmt2d^fl/fl^* mutants (21,664 genes).
